# Whole-Genome and Plasmid Comparative Analysis of Campylobacter jejuni from Human Patients in Toyama, Japan, from 2015 to 2019

**DOI:** 10.1128/spectrum.02659-22

**Published:** 2023-01-09

**Authors:** Daichi Morita, Hiroki Arai, Junko Isobe, Emi Maenishi, Takanori Kumagai, Fumito Maruyama, Teruo Kuroda

**Affiliations:** a Department of Microbiology, Graduate School of Biomedical and Health Sciences, Hiroshima University, Hiroshima, Japan; b Toyama Institute of Health, Toyama, Japan; c Section of Microbial Genomics and Ecology, The IDEC Institute, Hiroshima University, Hiroshima, Japan; South China Sea Institute of Oceanology

**Keywords:** *Campylobacter jejuni*, antimicrobial resistance, genomic epidemiology, pTet family plasmid, whole-genome sequencing

## Abstract

Campylobacter jejuni is a major causative agent of food poisoning, and increasing antimicrobial resistance is a concern. This study investigated 116 clinical isolates of C. jejuni from Toyama, Japan, which were isolated from 2015 to 2019. Antimicrobial susceptibility testing and whole-genome sequencing were used for phenotypic and genotypic characterization to compare antimicrobial resistance (AMR) profiles and phylogenic linkage. The multilocus sequence typing approach identified 37 sequence types (STs) and 15 clonal complexes (CCs), including 7 novel STs, and the high frequency CCs were CC21 (27.7%), CC48 (10.9%), and CC354 (9.9%). The AMR profiles and related resistant factors were as follows: fluoroquinolones (51.7%), mutation in quinolone resistance-determining region (QRDRs) (GyrA T86I); tetracyclines (27.6%), acquisition of *tet*(O); ampicillin (7.8%), harboring *bla*_OXA184_ or a promoter mutation in *bla*_OXA193_; aminoglycosides (1.7%), acquisition of *ant(6)-Ia* and *aph(3′)-III*; chloramphenicol (0.9%), acquisition of *cat*. The acquired resistance genes *tet*(O), *ant(6)-Ia*, *aph(3′)-III*, and *cat* were located on pTet family plasmids. Furthermore, three pTet family plasmids formed larger plasmids that incorporated additional genes such as the type IV secretion system. Sequence type 4526 (ST4526; 10.9%), which is reported only in Japan, was the most predominant, suggesting continued prevalence. This study reveals the sequences of the pTet family plasmids harbored by C. jejuni in Japan, which had been unclear, and the acquisition of the insertion sequences in a part of the pTet family plasmids. Because pTet family plasmids can be horizontally transmitted and are a major factor in acquired resistance in Campylobacter, the risk of spreading pTet that has acquired further resistance should be considered.

**IMPORTANCE** Campylobacter jejuni is among the major causes of enteritis and diarrhea in humans in many countries. Drug-resistant Campylobacter is increasing in both developing and developed countries, and in particular, fluoroquinolone-resistant Campylobacter was one of the species included on the priority list of antibiotic-resistant bacteria. Campylobacter drug resistance surveillance is important and has been conducted worldwide. In this study, we performed whole-genome analysis of Campylobacter jejuni isolated from diarrhea patients at a hospital in Toyama, Japan. This revealed the continued prevalence of Campylobacter jejuni ST4526, which has been reported to be prevalent in Japan, and the acquisition of resistance and virulence factors in the pTet family plasmids. The diversity of pTet family plasmids, the major resistance transmission factor, is expected to potentially increase the risk of Campylobacter. The usefulness of whole-genome sequencing in Campylobacter surveillance was also demonstrated.

## INTRODUCTION

Campylobacter is a Gram-negative commensal bacterium found in the gastrointestinal tract of many animals. Campylobacter jejuni are among the major causes of enteritis and diarrhea in humans in many countries, including Japan (1). Based on the annual food poisoning statistics compiled by the Ministry of Health, Labor and Welfare (MHLW) in Japan, since 2003, Campylobacter food poisoning has become the most prevalent bacterial foodborne disease (2). According to the Centers for Disease Control and Prevention (CDC) FoodNet surveillance program, in 2020 (http://www.cdc.gov/foodnet/surveillance.html), Campylobacter had a case incidence of 14.35 per 100,000 population among the causes of laboratory-confirmed bacterial foodborne illnesses in the United States. Livestock, such as poultry, cattle, and swine are asymptomatic reservoirs of pathogenic Campylobacter, and Campylobacter-contaminated food and water are major causes of foodborne illness worldwide (3, 4). In particular, consumption of poultry products is a risk factor for Campylobacter food poisoning (3, 4).

The use of antibiotics is usually not required for the treatment of uncomplicated campylobacteriosis; however, antibiotic therapy is necessary for severe systemic or chronic infections. For treatment of intestinal Campylobacter infections, macrolides are the drugs of choice. Fluoroquinolones are commonly chosen for empirical treatment of adults with suspected bacterial gastroenteritis, whereas tetracyclines are considered a second-line treatment and are also used for intestinal Campylobacter infections (1, 4–7). For the treatment of severe Campylobacter bacteremia and other systemic infections, intravenous aminoglycosides and carbapenems are considered a treatment option (8, 9). Throughout the years, the proportion of resistant Campylobacter has increased, and resistant Campylobacter has become a public health concern.

Macrolide resistance in Campylobacter remains low, but macrolide-resistant Campylobacter caused by 23S rRNA mutations and *emr*(B) have been reported (10–13). The rate of quinolone-resistant Campylobacter is continually increasing, although it varies from country to country, and in many countries fluoroquinolone-resistant Campylobacter is a concern (13–16). In addition, fluoroquinolone-resistant Campylobacter was listed as one of 12 priority pathogens by the World Health Organization (17). Resistance to fluoroquinolones in Campylobacter is primarily a point mutation in the quinolone resistance-determining region (QRDR) of GyrA (18–21). Tetracycline-resistant Campylobacter has been reported in many countries. Tetracycline resistance in Campylobacter is usually conferred by the ribosome protection protein *tet*(O), which prevents the binding of tetracycline to the ribosome (20, 22, 23). Acquisition of *tet*(O) is mainly due to the acquisition of the pTet family plasmid carrying the gene (24), although detection in chromosomes has occasionally been reported (25). The pTet plasmid first identified in C. jejuni 81-176, is a 45,205-bp plasmid containing *tet*(O) and capable of conjugative transfer between Campylobacter (26). The pTet family plasmids are the most prevalent plasmids in C. jejuni and Campylobacter coli (24). To date, several aminoglycoside resistance genes have been identified (14, 27–29), but the acquisition of aminoglycoside resistance genes in Campylobacter is not well understood. However, the presence of aminoglycoside resistance genes on pTet has been reported (20, 30–32), and pTet plays an important role in the horizontal spread of drug resistance in Campylobacter.

Whole-genome sequencing (WGS) is a highly informative and discriminative approach and can comprehensively analyze resistance factors as well as many properties such as virulence factors and phylogenetic relations. However, WGS of C. jejuni isolated from humans in Japan has been limited (33, 34). Although serotyping is a popular classification method for C. jejuni, recently, the number of untypeable strains has been increasing. Therefore, genome-based phylogenetic analysis using WGS can be used to investigate the prevalence of specific lineages. pTet family plasmids are known to be involved in resistance transmission in C. jejuni (24, 25, 35, 36), but the spread of pTet family plasmids in C. jejuni in Japan remains unclear. The analysis of resistance transfer factors by WGS analysis is important to control the spread of resistant bacteria. In this study, 116 C. jejuni strains isolated from humans in Toyama, Japan, from 2015 to 2019 were subjected to antimicrobial susceptibility tests and WGS to determine the prevalence of resistance and the resistance mechanism.

## RESULTS

### Phenotypic antimicrobial resistance analysis by antibiotic susceptibility testing.

**FIG 1:**
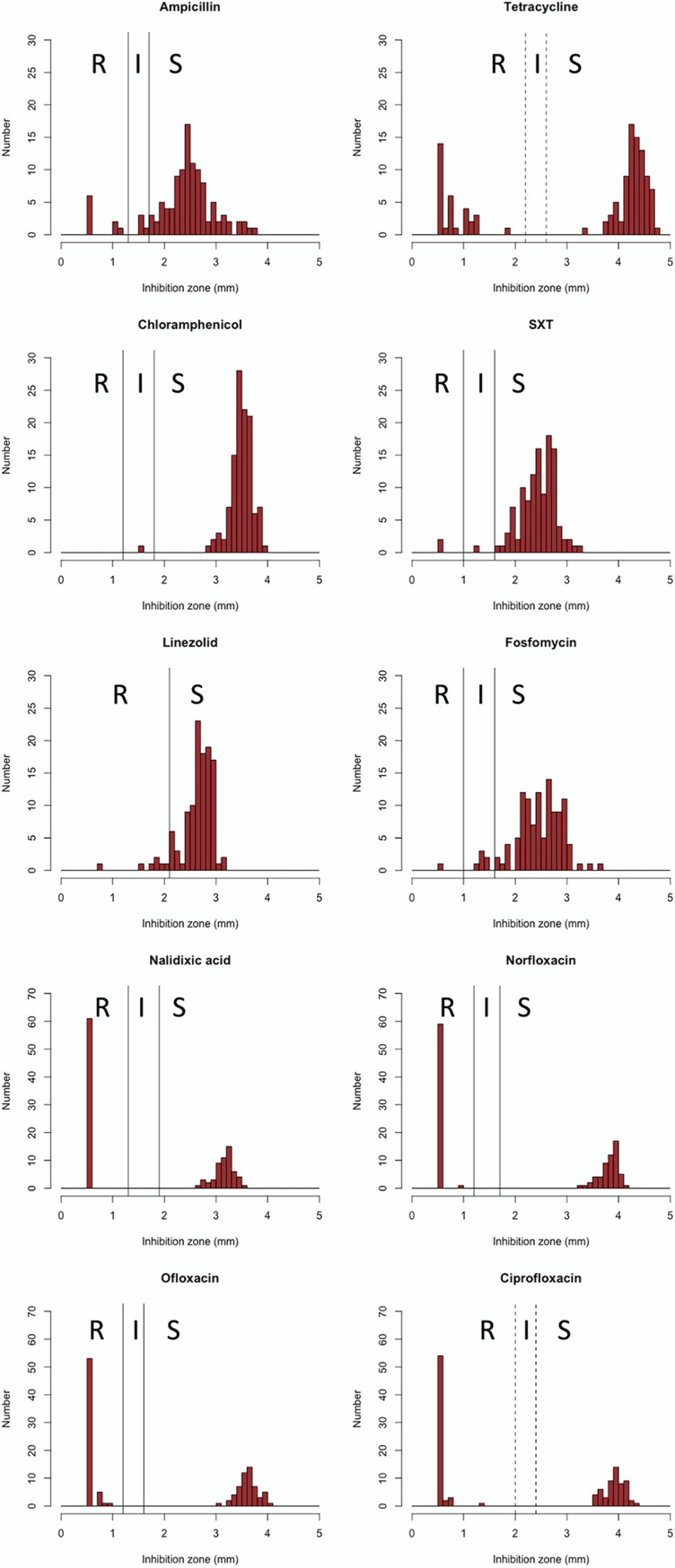
Distribution of antibiotic disc inhibition zone sizes for C. jejuni. Breakpoints of tetracycline and ciprofloxacin were determined according to the CLSI. In the case of antimicrobial agents for which there are no criteria in the CLSI (ampicillin, chloramphenicol, fosfomycin, linezolid, nalidixic acid, norfloxacin ofloxacin, and sulfamethoxazole-trimethoprim [SXT]), the criteria were based on the value of *Enterobacteriaceae* or Staphylococcus (linezolid only) spp.

Antibiotic susceptibility testing was confirmed using 24 antibiotics with the disc diffusion method (see Table S1 in the supplemental material). Breakpoints were determined according to the Clinical and Laboratory Standards Institute (CLSI) guidelines, and if the CLSI had no criteria, then the criteria were based on the value for *Enterobacteriaceae* or Staphylococcus spp. (linezolid only). Campylobacter exhibits intrinsic resistance to novobiocin, bacitracin, trimethoprim, rifampicin, most of the β-lactams, vancomycin, and polymyxin/colistin (11, 37, 38). In this study, all C. jejuni isolates were also resistant to novobiocin, rifampicin, colistin, and some β-lactams, such as cloxacillin, cefmetazole, and aztreonam. Except for the intrinsic resistance, fluoroquinolone (norfloxacin, ciprofloxacin, and ofloxacin) (51.7%, 60/116) and tetracycline (27.6%, 32/116) resistance were frequently observed. Rarely, ampicillin (7.8%, 9/116), streptomycin (0.9%, 1/116), kanamycin (0.9%, 1/116), sulfamethoxazole-trimethoprim (1.7%, 2/116), linezolid (3.4%, 4/116), and fosfomycin (0.9%, 1/116) resistance and chloramphenicol (0.9%, 1/116) intermediate resistance were also observed (Fig. 1). No strains resistant to cefaclor, imipenem, erythromycin, streptomycin amikacin, arbekacin, or gentamicin were identified.

### Genetic antimicrobial resistance analysis by whole-genome sequencing.

**FIG 2:**
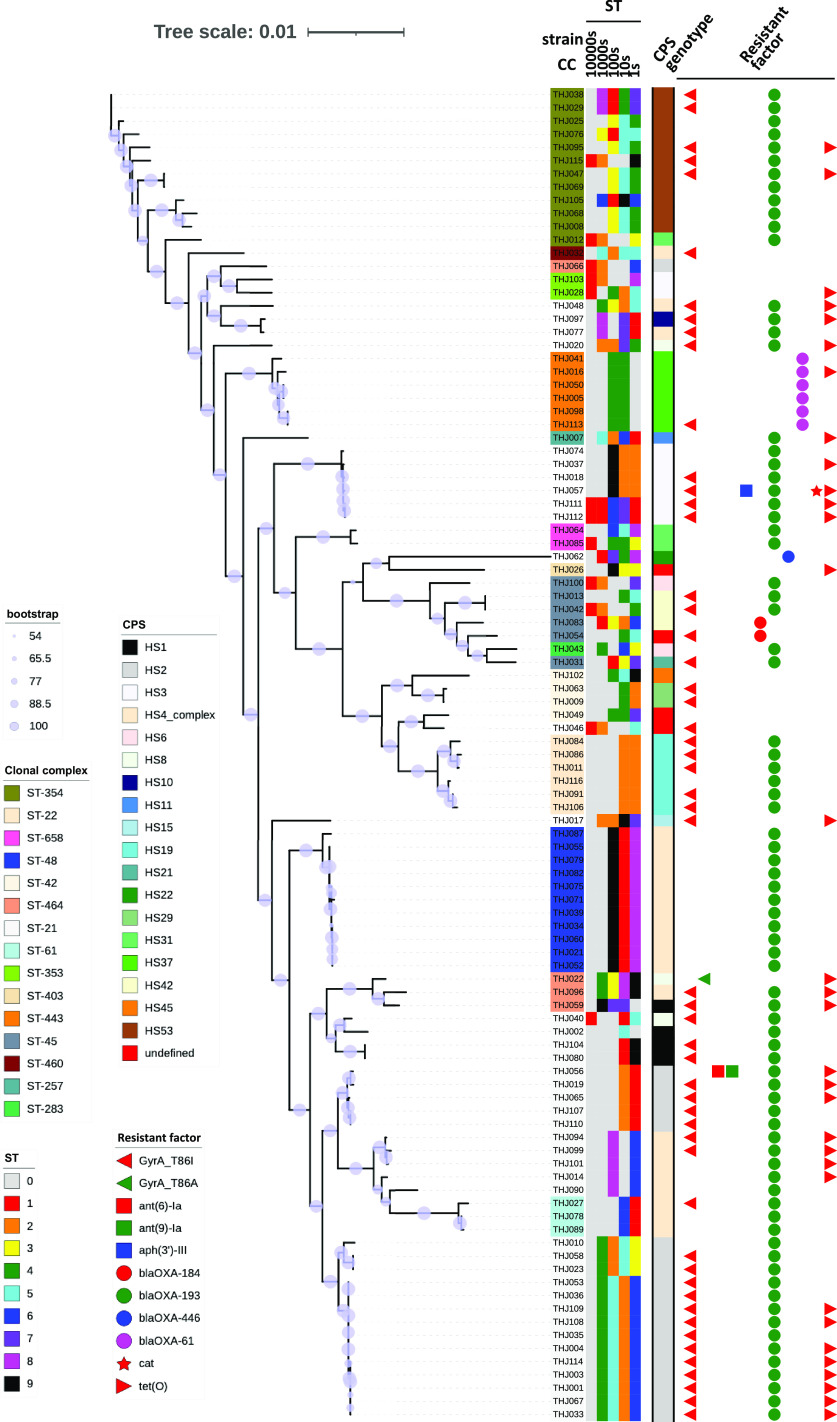
Maximum likelihood phylogenetic tree and distribution of the genotype (MLST, CPS genotype, and antimicrobial resistance [AMR] genes) of the 101 C. jejuni isolates. Colored bar on the right, from inner to outer: 1st to 5th represent ST (sequence type), 6th represents CPS (capsule polysaccharide) genotyping pattern, and 7th to 16th represent isolates harboring genetic determinants of resistance. The names of the isolates are color-coded according to CC (clonal complex). The sizes of the purple circles at the nodes of phylogenetic tree indicate the magnitude of bootstrap values.

Among the 116 isolates, 101 strains were isolated within a week and subjected to WGS, except for strains with similar resistance profiles. Molecular analysis of C. jejuni predicted resistance factors that correlated with the phenotype corresponding to the phenotype of antimicrobial resistance (AMR) (Table S1, Fig. 2).

All fluoroquinolone-resistant isolates had detected a mutation in the quinolone-resistant determining region (QRDRs), *gyrA* (T86I). One nalidixic acid-resistant, fluoroquinolone-sensitive strain was found. This strain had a mutation in *gyrA* (T86A).

In tetracycline-, streptomycin-, kanamycin-, and chloramphenicol-resistant isolates, the presence of *tet*(O), *ant(6)-Ia*, *aph(3′)-III*, and *cat* genes, respectively, was observed. Although the high prevalence of the class D β-lactamases *bla*_OXA-61_, *bla*_OXA-184_, *bla*_OXA-193_, and *bla*_OXA-446_ was identified, only 9 ampicillin-resistant isolates were found. Ampicillin-resistant isolates had *bla*_OXA-184_ or the G → T transversion in the *bla*_OXA-193_ promoter region (57 bp upstream of the annotated start codon). Although one ampicillin-resistant strain harbored *bla*_OXA-193_, there was no mutation in the promoter region, so the cause of the resistance was unknown. This mutation in the promoter has been reported to be associated with high-level expression of *bla*_OXA-61_ in C. jejuni (39).

Sulfamethoxazole-trimethoprim is a combination of two antibiotics that act synergistically against a wide variety of bacteria. Although trimethoprim acts as a specific inhibitor of bacterial dihydrofolate reductase (FolA), Campylobacter is innately resistant to trimethoprim due to the lack of FolA (40). Therefore, this resistance may be mediated by resistance to sulfamethoxazole. In general, the sulfamethoxazole resistance mechanism has been reported as the acquisition of the low-affinity alternative dihydropteroate synthase (DHPS) genes, such as *sul1*, *sul2*, and *sul3*, and mutations in the chromosomal DHPS gene, *folP* (41). The alternative DHPS genes *sul1*, *sul2*, and *sul3* were not detected in the two sulfamethoxazole-trimethoprim-resistant isolates, THJ056 and THJ103. Compared with the susceptible isolates, no unique mutation in *folP* up to 200 bp upstream was present in THJ103, while a 27-bp insertion in *folP* was found in THJ056. It has been reported that substitution and insertion in 64th proline of FolP, such as in Escherichia coli, Neisseria meningitidis, and Haemophilus influenzae, are related to sulfamethoxazole resistance (42–44). The 166th proline of C. jejuni corresponds to the 64th proline of E. coli, and the amino acid insertion was located near this point, S168_R169insVYCGKEEEF (Fig. S1). In addition to the insertion, the G167K mutation was also observed, and this may be responsible for sulfamethoxazole resistance.

In general, linezolid resistance is mediated by point mutations in domain V of the 23S rRNA (45). In addition, *optrA*, encoding an ATP-binding cassette F (ABC-F) protein that confers resistance to oxazolidinones and phenicols, has been found in Campylobacter (46). However, in this study, none of these resistance factors were predicted in linezolid-resistant isolates.

Several mechanisms of fosfomycin resistance in Gram-negative bacteria have been reported, including target modification, expression of antibiotic-degrading enzymes, reduced uptake, and rescue of the UDP-MurNAc biosynthetic pathway (47). In Campylobacter, the fosfomycin-inactivating enzyme gene *fosX^CC^* was reported (48). However, *fosX^CC^* was not detected in the fosfomycin-resistant isolate.

### Molecular characterization of the pTet family plasmids.

**FIG 3:**
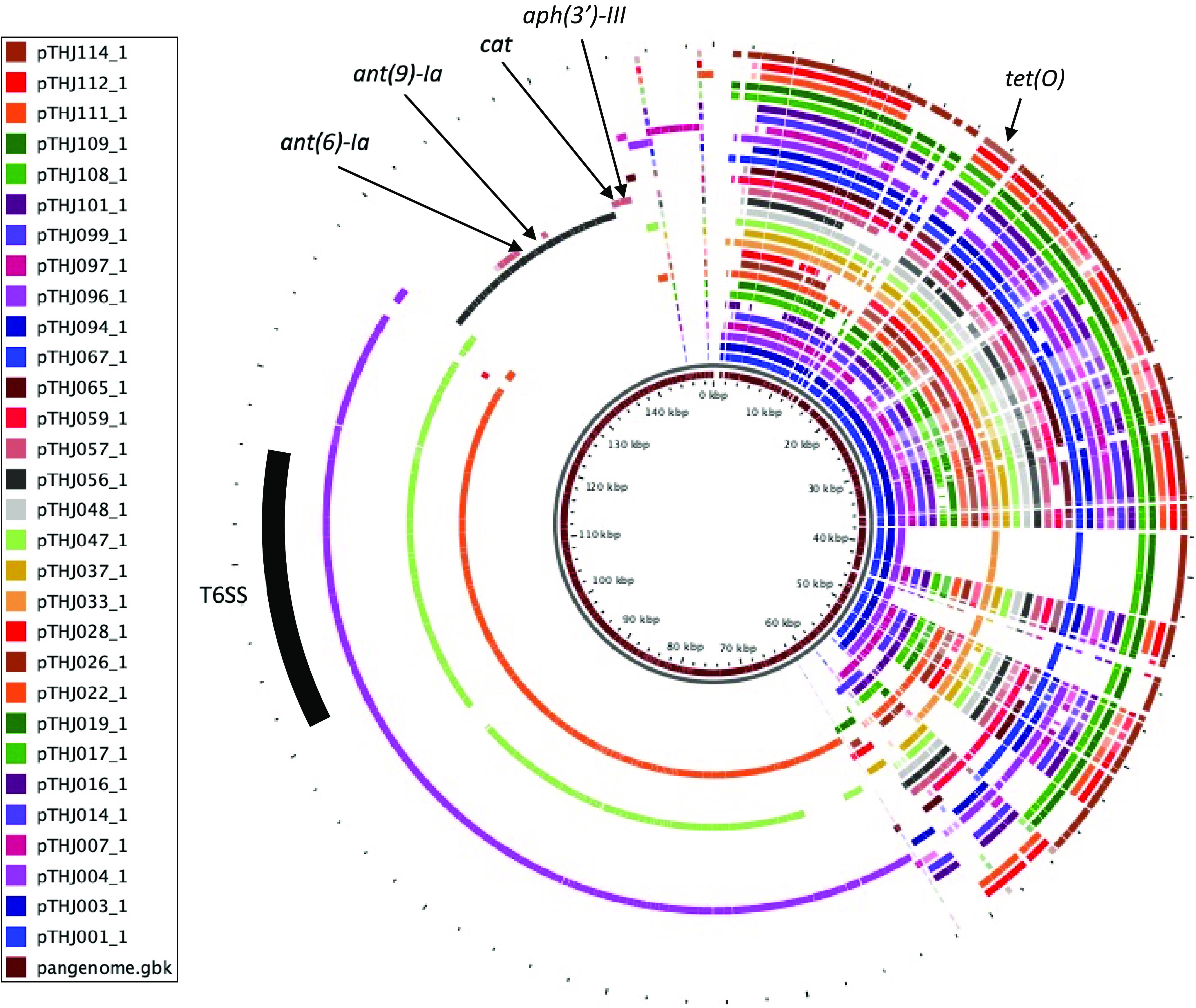
Pangenome of pTet plasmids in isolates detected by WGS. The innermost circle shows the pangenome (brown), and outer circles indicate the plasmid.

**FIG 4:**
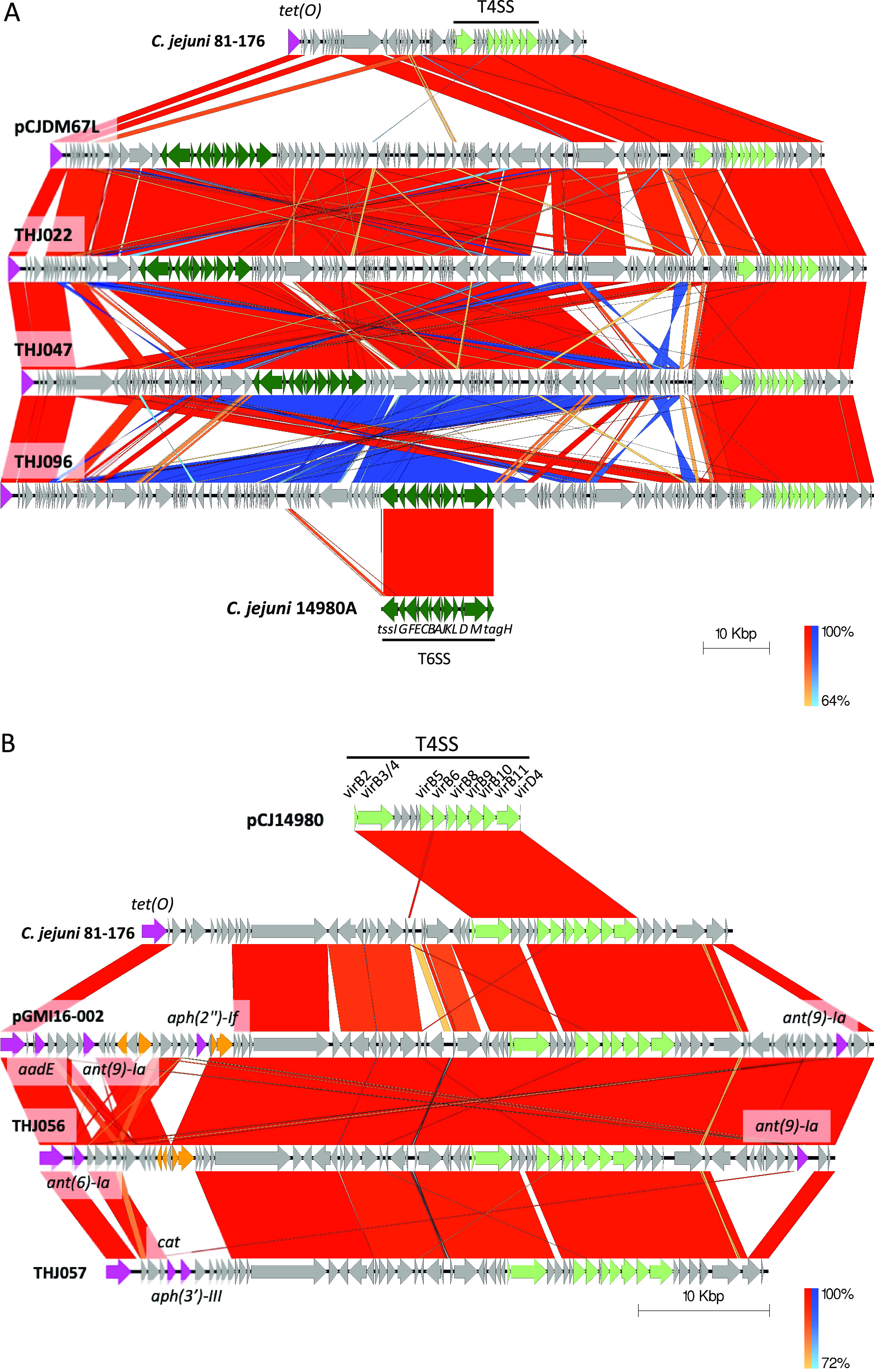
Plasmid sequence comparison. (A) Sequence alignment of pTet plasmid inserted with extra DNA containing type VI secretion system (T6SS)-related genes of isolates (THJ021, THJ047, and THJ096) with the reference pTet plasmid of C. jejuni 81-176 (NC_008790.1) and pCJDM67L of C. jejuni strain OD267 (CP014745.1). (B) Sequence alignment of pTet plasmid inserted with extra resistant genes of isolates (THJ055 and THJ056) with the reference pTet plasmid of C. jejuni 81-176 (NC_008790.1) and pGMI16-002 of C. jejuni strain CFSAN054107 (CP028186.1). Vertical blocks between sequences indicate regions of shared similarity shaded according to BLASTn (red for matches in the same direction and blue for inverted matches). Coding sequences (CDS) are represented by colored arrows. CDSs are characterized by their functions as follows: resistance genes (pink), transposons/integrases (orange), type IV secretion system (T4SS) genes (pale green), T6SS genes (dark green), and others (gray). The outer scale is marked in 10 kb. The T4SS and T6SS regions were identified by reference to C. jejuni strain 14980A plasmid pCJ14980 (CP017030.1) and a C. jejuni strain 14980A chromosome (CP017029.1).

The *tet*(O) gene was present in the plasmids except for in one strain, and the plasmid belonged to the pTet family, the most prevalent plasmid type in Campylobacter (24, 49). Pangenome analysis showed that the plasmids had high similarity; however, a few plasmids were observed with extra DNA length that included some genes (Fig. 3). Three isolates had plasmids (THJ022, THJ047, and THJ096) that were similar to pCJDM67L and pCJDM202 found in C. jejuni and had more than 60 kbp extra DNA containing type VI secretion system (T6SS)-related genes identified in diverse species of Gram-negative bacteria and act to kill competing bacteria via a bacteriophage-like invasion and the injection mechanism (Fig. 4A) (50). The other two isolates had plasmids (THJ056 and THJ057) with resistance genes other than *tet*(O) were found to be similar to plasmid pGMI16-002 (CP028186.1) from the C. jejuni strain CFSAN054107; one had *ant(6)-Ia* and *ant(9)-Ia*, and the other had *aph(3′)-III* and *cat* (Fig. 4B) (51).

### Sequence types (STs), phylogenetic relatedness, and pangenome.

**FIG 5:**
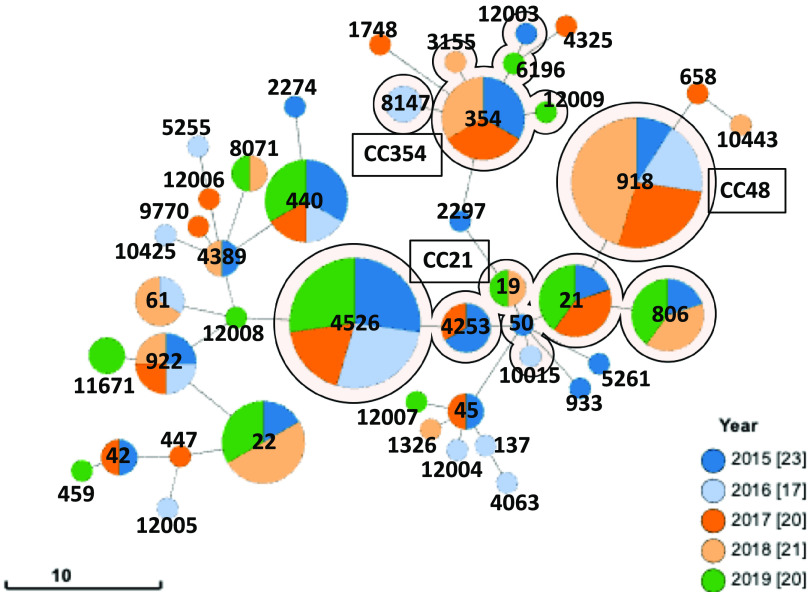
Minimum spanning tree of C. jejuni sequence types (STs) according to MLST profiles. Each ST is represented as a circle, with the size of the circle proportional to the number of isolates, and the inside colors indicate isolate years. The branch length represents the allelic distance. Background shading highlights typical clonal complexes (CCs) in this data set.

Multilocus sequence typing (MLST), based on the variation among the seven housekeeping alleles defined as sequence types (STs) and clonal complexes (CCs), is used for phylogenetic and epidemiological analyses. In the present study, the 101 isolates matched 37 STs from the reference pubMLST database, and 7 isolates did not match the database due to an untypeable locus combination or alleles which represent novel STs. The 37 STs were linked to 15 CCs (Table S1, Fig. 2 and 5).

The isolates were distributed widely across different CCs, with the majority belonging to CC21 (27.7%, 28/101), CC48 (10.9%, 11/101), and CC354 (9.9%, 10/101). The distribution of STs throughout the years of isolation was not characterized, and there were no STs identified permanently during the 5 years of this study, suggesting that food poisoning by C. jejuni is a sporadic event in Toyama, Japan.

Comparative genomic analysis was applied for a more detailed phylogenetic analysis of the 101 isolates subjected to WGS (Fig. 2). The results of the phylogenetic analysis and MLST analysis were in good agreement. Within some STs, such as ST918, ST4526, and ST922 to ST11671, genomic diversity among strains is quite low, suggesting that single clones are prevalent. The pTet family plasmids were associated with several STs, 16 of which were linked to 7 CCs, and the ST types were phylogenetically distinct.

Penner serotyping, which is also called heat-stable (HS) serotyping has been the principal method to classify different Campylobacter isolates (52). Campylobacter capsule polysaccharide (CPS) is the principal serodeterminant of Penner serotyping (53). Currently, 47 HS serotypes are recognized in C. jejuni, which correlates with the variation in CPS. Recently, HS serotyping has become untypeable in many cases, and gene-based HS classification using the PCR method for CPS loci has been proposed as an alternative method (54). In this study, HS types were classified by *in silico* PCR.

Among 116 isolates, 17 serotypes were identified, including untypeable isolates. Although ST and HS types were correlated, half of the isolates were untypeable (*n* = 63) in HS serotyping, so HS serotyping was not sufficient to classify C. jejuni in this study (Table S1). The 101 strains that were subjected to WGS were analyzed for the regions amplified by gene-based typing of the CPS loci, as proposed by Poly et al. (Table S1, Fig. 2) (54). This typing method classified most of the strains (96.0%, 97/101), including those that were untypeable in HS serotyping, and the method was consistent with HS serotyping, except in 4 isolates: THJ054 (HS38 and undefined HS in HS serotyping and gene-based typing), THJ056 (HS22 and HS2 in HS serotyping and gene-based typing), THJ113 (HS3 and HS37 in HS serotyping and gene-based typing), and THJ116 (HS3 and HS19 in HS serotyping and gene-based typing). The predominant serotype was HS4 complex (*n* = 21), followed by HS2 (*n* = 20) and HS53 (*n* = 11). The gene-based typing results are consistent with those of comparative genome analysis, and this method can be reliable in estimating the HS serotypes of C. jejuni.

## DISCUSSION

WGS is a highly informative and discriminative approach and has become a major tool in the investigation of the epidemiology of bacteria. WGS of C. jejuni isolated from humans in Japan has been limited, and this study analyzed resistance factors and phylogenetic analysis by WGS (33, 34). In addition, we determined the sequences of pTet family plasmids, a major resistance transfer factor of Campylobacter, but unclear in Japan, and performed comparative analysis.

In this study, the major AMR in C. jejuni, excluding intrinsic resistance, was 51.7% for fluoroquinolone resistance and 27.6% for tetracycline resistance. Asakura et al. showed that the resistance rates of C. jejuni in humans and chickens were 36% for ciprofloxacin and tetracycline in 2005 to 2006 and were 64% for ciprofloxacin and 42% for tetracycline, respectively in 2010 to 2011 (55). Yamada et al. revealed that the ciprofloxacin resistance rates in the two periods 2000 to 2008 and 2009 to 2017 were 34.9% and 41.9%, respectively (56). The results of our study were in agreement with these previous studies. The main mechanisms of quinolone resistance involving C. jejuni have been well studied. Among them, mutations of QRDRs, in the DNA gyrase (consisting of GyrA and GyrB) and topoisomerase IV (consisting of ParC and ParE), are the targets of quinolones, are the most common and are found in almost all microorganisms. C. jejuni has been reported to lack *parCE* (19, 57–59), and a T86I in GyrA confers a high level of resistance to fluoroquinolones (21). It has also been reported that T86A in GyrA is sensitive to fluoroquinolones but confers resistance to nalidixic acid (60). In this study, all the high-level quinolone-resistant strains had T86I in GyrA, whereas the low-level quinolone-resistant strains had T86A in GyrA, which is consistent with previous studies.

The tetracycline resistance was due to the acquisition of *tet*(O), as in previous reports of tetracycline-resistant C. jejuni, and was mostly due to the acquisition of pTet family plasmids (24, 25, 35, 36). The pTet family plasmids are conjugative plasmids and are the most frequently found plasmids in C. jejuni and C. coli (61). The sequences are highly conserved, although additional gene insertions have been reported sporadically around the world (20, 24). However, detailed characterization of the sequences harbored by Campylobacter in Japan has been unclear. In this study, the complete sequences of 30 pTet family plasmids were characterized, and 25 plasmids had nearly identical structures with no additional inserted sequences. The strains harboring pTet family plasmids were observed in a wide range of STs, suggesting that they are widely distributed among C. jejuni in Japan. However, the same ST is also divided in its harboring status, and plasmid acquisition and loss may occur frequently. The two plasmids with inserted resistance genes of AME and *cat* were identical to the insertion positions in the previously reported plasmids with resistance gene insertions (20, 61). The three pTet plasmids with large inserted sequences including T6SS were also observed. Similar pTet family plasmids have been reported in several cases of C. jejuni and C. coli, and this plasmid acquisition was reported to increase *in vitro* hemolytic activity (50). The inserted sequences and insertion sites were nearly identical, but inversions and rearrangements were observed. Although few such plasmids were reported, the insertion of new resistance genes into pTet family plasmids, which are widely harbored in C. jejuni, suggests that pTet family plasmids may enhance the multidrug resistance and facilitate survival and virulence of C. jejuni.

C. jejuni is inherently resistant to most β-lactams but is susceptible to some β-lactams, such as ampicillin. In this study, *bla*_OXA_ was detected in almost all strains, but ampicillin resistance was confirmed in only a few. In the ampicillin-resistant strains, a mutation was found in the promoter region, as in previous reports, suggesting that this promoter mutation increased the expression of *bla*_OXA_ (39). Most C. jejuni harbor *bla*_OXA_, suggesting that they are potentially resistant to ampicillin.

On the other hand, macrolide-resistant strains have been reported as rare in Japan (33, 55, 56, 62), and no macrolide-resistant strains were found in this study, suggesting that macrolide resistance remains rare in Japan.

The 101 isolates were classified into 37 STs, including 7 novel STs, indicating that clinical isolates of C. jejuni exhibit high molecular diversity. CC21 was the most common CC type (28.7%, 29/101). Among CC21 isolates, ST4526 was predominant (11/29) and showed high resistance rates to fluoroquinolones (11/11) and tetracycline (8/11). ST4526, which was first found in Japan in 2012, is a Japan-specific lineage. Ohishi et al. also reported that ST4526 was the most dominant among human and chick isolates in Japan from 2007 to 2014 (34). Furthermore, Yamada et al. revealed that ST4526, while not isolated in 2000 and 2008, became the dominant ST in 2017 (56). These results suggest that ST4526 is still spreading in Japan. CC48, the second most common CC type, was detected only in ST918, which is often isolated from poultry in Japan, and was equal in number to ST4526. The global representative of CC21 is ST50, the higher ST type of C. jejuni, which has been isolated in Australia, Europe, North America, and Asia (14, 63). Although ST50 has been equally isolated with ST4526 in previous reports from Japan (33, 34), ST50 was isolated less frequently in our study. Since ST4526 has been reported to outcompete ST21, ST50, and ST53 in colony-forming ability in the chicken intestine (55), ST4526 might be replacing ST50 and others that were previously predominant. Further monitoring of the prevalent strains of C. jejuni in Japan is required.

In the serotyping of C. jejuni, the Lior method, based on thermophilic antigens, and the Penner method, based on thermostable antigens, are internationally recognized (52, 64). The major antigenic determinant in the Penner serotyping method is CPS, a cell surface molecule that affects bacteriophage infectivity (65), colonization of chickens (66), invasion of human epithelial cells (61, 67), and host immune responses (38). The CPS gene cluster contains multiple phase-variant genes interrupted by poly-G tracts, resulting in the unstable phenotype of the Penner serotype due to the on/off expression of CPS genes due to phase variation. For this reason, Penner serotyping frequently results in untypeable strains. Recently, a method to determine the Penner genotype by PCR typing of CPS gene clusters (Penner genotyping method) was reported (54). The genotyping method was able to type most of the strains that were untypeable by the current serotyping method. In addition, phylogenetic classification by core genome analysis correlated well with the genotyping, supporting the reliability of the genotyping results. The genotyping method is considered an effective alternative to the Penner serotyping method because of its high typing rate and correlation with existing serotyping methods, although some issues need to be investigated further, such as the expression of CPS genes.

These results suggest that the major drug resistances of C. jejuni clinical isolates are fluoroquinolones and tetracycline, and macrolides remain susceptible. In addition, strain ST4526, belonging to CC21, was highly resistant to fluoroquinolones and tetracycline, suggesting that it is mainly spreading in Japan. Moreover, the acquisition of resistance genes and virulence genes was confirmed in a part of the pTet family plasmid transmitting tetracycline resistance, and the expansion of antimicrobial-resistant C. jejuni is expected in the future. It is important to monitor the spread of C. jejuni resistance by antimicrobial susceptibility and molecular analysis.

## MATERIALS AND METHODS

### Bacterial isolates.

C. jejuni strains were isolated from patients with gastrointestinal symptoms and identified by the Toyama Institute of Health in Toyama, Japan, from 2015 to 2019 (Table S1). A total of 116 isolates were used, 112 of which were isolated at one hospital (JA Toyama Kouseiren Takaoka Hospital) in Toyama Prefecture, and 4 of which were collected by the Toyama City Public Health Center from a food poisoning outbreak in Toyama Prefecture. The strains were stored at −80°C in brain heart infusion broth containing 15% glycerol. Among the 112 isolates from the hospital, 26 were isolated in 2015, 19 were isolated in 2016, 22 were isolated in 2017, 28 were isolated in 2018, and 17 were isolated in 2019. Among the 4 isolates from the Public Health Center, 1 was isolated in 2016 and 3 were isolated in 2019. All strains were evaluated for antimicrobial susceptibility using the disc diffusion method, and 101 strains were subjected to WGS.

### Antimicrobial susceptibility testing.

The disc diffusion methodology was based on National Committee for Clinical Laboratory Standards (68) recommendations. The disc content was as follows: ampicillin, 10 μg; cloxacillin, 1 μg; cefaclor, 30 μg; cefmetazole, 30 μg; imipenem, 10 μg; aztreonam, 30 μg; tetracycline, 30 μg; erythromycin, 15 μg; streptomycin, 10 μg; kanamycin, 30 μg; amikacin, 30 μg; arbekacin 30 μg; gentamicin, 10 μg; nalidixic acid, 30 μg; norfloxacin, 10 μg; ofloxacin, 5 μg; ciprofloxacin, 5 μg; sulfamethoxazole-trimethoprim, 23.75/1.25 μg; linezolid, 30 μg; colistin, 10 μg; chloramphenicol, 30 μg; fosfomycin, 50 μg; rifampicin, 5 μg; novobiocin, 30 μg. All the discs were Sensi-Discs (Becton, Dickinson). The isolates were grown on TSA II 5% sheep blood agar (Becton, Dickinson) at 42°C for 24 h under microaerobic conditions (AnaeroPack-MicroAero; Mitsubishi Gas Chemical Company, Inc., Japan). Inoculum was prepared to equal 0.5 McFarland standard of direct colony suspension. Antibiotic sensitivity testing was conducted using Mueller-Hinton agar with 5% sheep blood (Becton, Dickinson); the inoculum was spread over the plate three times with a sterile cotton swab and incubated at 42°C for 24 h under microaerobic conditions.

### Whole-genome sequencing, genome assembly, and antimicrobial resistance analysis.

DNA was extracted from single-colony cultures (Monarch Genomic DNA purification kit, NEB). Short-read libraries were prepared based on the NEBNext Ultra II FS DNA library prep kit with sample purification beads (NEB) and sequenced using an Illumina MiSeq v3 device (150 cycles). Long-read libraries were prepared based on the rapid barcoding kit (Nanopore) and sequenced using Oxford Nanopore MinION technology.

Paired-end 150-bp FASTQ files were passed through the Bactopia 1.7.1 workflow to assess data quality, assemble contigs, and call *in silico* MLST (69). In the long-read assembly, the long reads with greater than 400× coverage were randomly downsampled to a coverage of 400× using rasusa v0.6.1 (parameters: –coverage 400 –genome-size 1.7 Mb) (70). The long reads were filtered using NanoFilt v2.8.0 (-l 3000 –headcrop 75) (71). Flye v2.9 (72) was used, and the assembled sequences were polished using Illumina reads with Pilon v1.24 (73) with default parameters. Plasmids constructed by tandem assembly of identical small plasmid sequences were manually resolved.

The assembled genomes were then passed through a bioinformatic pipeline using BLAST techniques to identify AMR genes and AMR-associated point mutations using the programs STARAMR v0.7.2 (https://github.com/phac-nml/staramr) and ABRicate. STARAMR databases used PointFinder v050218 (database date on 7 January 2022), ResFinder v050218.1 (database date 6 October 2021), MLST v2.9 (https://github.com/tseemann/mlst), and the PlasmidFinder database (database date on 7 January 2022) (74–77). ABRicate (https://github.com/tseemann/abricate) used the ResFinder, NCBI, CARD, and MEGARes databases (last updated on 3 February 2022) (77–80). FolA alignment was performed using the online version of MAFFT v7 (https://mafft.cbrc.jp/alignment/server/) (81, 82). Minimum spanning trees were generated and visualized in GrapeTree (83).

### Plasmid comparison analysis.

Pangenome analysis for plasmids from our laboratory was conducted using the GView server (https://server.gview.ca/) (84). In the GView server, BLAST analysis (nucleotide) was carried out using GenBank files of plasmid sequences with an E value of <1e-10, alignment length cutoff value of 100, and percent identity cutoff value of 80. The comparison of plasmids in which gene insertion was detected with C. jejuni 81-176 plasmid pTet (NC008790.1) as a reference was visualized using Easyfig v2.2.5 (85).

### Genome comparison and phylogenetic analysis.

To identify the genus core genome, we used Panaroo v1.2.9 (86) to generate a gene presence-absence matrix with the default settings and the -a core flag to generate a core gene alignment. A core gene phylogeny was constructed from the core gene alignment using the IQ-Tree software v2.0.3 (87) with the GTR substitution model and ultrafast bootstrapping (1,000 bootstraps). Phylogenetic tree visualization was conducted using the Interactive Tree of Life v6.5 (iTOL) (https://itol.embl.de) (88).

### *In silico* identification of Penner genotype.

The presence of the specific CPS sequences for a particular serotype was determined by performing a local stand-alone BLAST search using a database encompassing the nucleotide sequences of the CPS genotyping multiplex PCR amplification region (54). Primers and their respective PCR product sizes are listed in Table S2.

### Data availability.

The genome sequences analyzed in this study are available under BioProject accession number PRJDB13586. The accession numbers and BioSample identifiers (IDs) are listed in Table S3 in the supplemental material.
