# Supplementary material for: Whole-Genome and Plasmid Comparative Analysis of Campylobacter jejuni from Human Patients in Toyama, Japan, from 2015 to 2019
Source: Microbiol Spectr. 2023 Jan 9;11(1):e02659-22. doi: 10.1128/spectrum.02659-22 (PMC9927224; doi:10.1128/spectrum.02659-22)
Supplement: Supplemental file 1 — Fig. S1. Download spectrum.02659-22-s0001.pdf, PDF file, 0.1 MB [file spectrum.02659-22-s0001.pdf]

Figure S1

```
b3177   1     MK------------------------------------------------------------------------------

Cj0585  1     MKFFKINPNTDFNLLCSFINPHKMGQKIMSEKTKIHFIFIKDISTPAANILKQDALRVGAELITHKEVITAKITHSNALL
THJ056  1     MKFFKINPNTDFNLLCSFINPHKMGQKIMSEKTKIHFIFIKDISTPAANILKQDALRVGAELITHKEVITAKITHSNTLL
THJ103  1     MKFFKINPNTDFNLLCSFINPHKMGQKIMSEKTKIHFIFIKDISTPAANILKQDALRIGAELITHKEVITAKITHSNALL
                                                                       *                  *

b3177   3     -------------------------LFAQGTSLDLSHPHVMGILNVTPDSFSDGGTHNSLIDAVKHANLMINAGATIIDV

Cj0585  81    MASKEQIQKLVVKEKLQDFGLKNLALFLQKDFLKPKKAELMAVINVNEDSF-NAKSRVSEEDFEKRLNDFLALKPEYIDI
THJ056  81    MATKEQIQKLINKEKLQDFGLKNLARFLENGFSKPKQAELMAVINVNEDSF-NADSRVSYKDFEERLNEILALNPEYIDI
THJ103  81    MASKEQIQKLIAKEKLQDFGLKNLALFLQKDFLKPKKAELMAVINVNEDSF-NAKSRVSEEDFEKRLNDFLALKPAYIDI
                *       **             *  *** *   *                 *    **   *   **     *

b3177   58    GGESTRPGA---------AEVSVEEELQRVIPVVEAIAQRF---EVWISVDTSKPEVIRESAKVGAHIINDIRSLSEPGA

Cj0585  160   GAVSSRPGS---------EYCGKEEEFKRLKKVLDLIYEKNYYEQAIFSLDSFDEYCLEYALNKGFKLINDITSLRNLNL
THJ056  160   GAVSSRPKSVYCGKEEEFRYCGKEEEFRRLKNVLDLIYDKNYYEKAIFSLDSFDEYCLEYALNKGFKFINDISSLRNLNL
THJ103  160   GAVSSRPGS---------EYCGKEEEFKRLKKVLDLIYEKNYYEQAIFSLDSFDEYCLEYALNKGFKLINDITSLRNLNL
                     * **********        *          *     *                      *    *

b3177   126   LEAAAETGLPVCLMHMQGNPKTMQEAPKYDDVFAEVNRYFIEQIARCEQAGIAKEKLLLDPGFGFGKNLSHNYSLLARLA

Cj0585  231   AKLASKYGAKYCLMHMQNNPNNMQDNPFYEDLLDEMTLFFKEKLELLESFGVKES--ILDVGIGFGKSAGHNMILIKHLE
THJ056  240   AKLASKYNAKYCLMHMQNDPLTMQDDPRYDDLLDEMSSFFKEKLEILDTLGVKES--ILDVGIGFGKSAEHNMILIKHLE
THJ103  231   AKLASKYEAKYCLMHMQNNPNNMQDNPSYEDLLDEMTLFFKEKLELLESFGVKES--ILDVGIGFGKSAGHNMILIKHLE
                     *            **     * *      **       * ***                   *

b3177   206   EFHHFNLPLLVGMSRKSMIGQLLNVGPSERLSGSLACAVIAAMQGAHIIRVHDVKETVEAMRVVEATLSAKENKRYE

Cj0585  309   HFLQFNKPLLIGASRKSVINAYFQSEIKDRLAGTLYLHLKAFENGASIIRVHDLYEHKQLFALAQAMDNIG-----V
THJ056  318   HFLQFKKPLLIGASRKSVINAYYESEVKDRLAGTLYLHLKAFENGASIIRVHDLYEHKQLFAMANAMQSIG-----V
THJ103  309   HFLQFNKPLLIGASRKSVINAYFQSEIKDRLAGTLYLHLKAFENGASIIRVHDLYEHKQLFALAQAMDNIG-----V
                                        *                                   * *  **
```

Figure S1 **Alignment of the FolP amino acid sequences of the sulfamethoxazole-trimethoprim resistant isolates.**

FolP (b3177) in *E. coli* K-12 MG1655 (NC_000913.3) and FolP (Cj0585) in *C. jejuni* subsp. *jejuni* NCTC 11168 = ATCC 700819 (NC_002163.1) were used as references. FolP in sulfamethoxazole-trimethoprim resistant isolates, THJ055 and THJ105, were aligned with the references.
